# Quantification of Behavioral Deficits in Developing Mice With Dystonic Behaviors

**DOI:** 10.3389/dyst.2022.10494

**Published:** 2022-09-08

**Authors:** Meike E. Van Der Heijden, Jason S. Gill, Alejandro G. Rey Hipolito, Luis E. Salazar Leon, Roy V. Sillitoe

**Affiliations:** 1 Department of Pathology & Immunology, Baylor College of Medicine, Houston, TX, United States; 2 Jan and Dan Duncan Neurological Research Institute at Texas Children’s Hospital, Houston, TX, United States; 3 Department of Pediatrics, Baylor College of Medicine, Houston, TX, United States; 4 Department of Neuroscience, Baylor College of Medicine, Houston, TX, United States; 5 Development, Disease Models & Therapeutics Graduate Program, Baylor College of Medicine, Houston, TX, United States

**Keywords:** dystonia, cerebellum, behavior, development, neurodevelopmental disorders

## Abstract

Converging evidence from structural imaging studies in patients, the function of dystonia-causing genes, and the comorbidity of neuronal and behavioral defects all suggest that pediatric-onset dystonia is a neurodevelopmental disorder. However, to fully appreciate the contribution of altered development to dystonia, a mechanistic understanding of how networks become dysfunctional is required for early-onset dystonia. One current hurdle is that many dystonia animal models are ideally suited for studying adult phenotypes, as the neurodevelopmental features can be subtle or are complicated by broad developmental deficits. Furthermore, most assays that are used to measure dystonia are not suited for developing postnatal mice. Here, we characterize the early-onset dystonia in *Ptf1a*^*Cre*^*;Vglut2*^*fl/fl*^ mice, which is caused by the absence of neurotransmission from inferior olive neurons onto cerebellar Purkinje cells. We investigate motor control with two paradigms that examine how altered neural function impacts key neurodevelopmental milestones seen in postnatal pups (postnatal day 7–11). We find that *Ptf1a*^*Cre*^*;Vglut2*^*fl/fl*^ mice have poor performance on the negative geotaxis assay and the surface righting reflex. Interestingly, we also find that *Ptf1a*^*Cre*^*;Vglut2*^*fl/fl*^ mice make fewer ultrasonic calls when socially isolated from their nests. Ultrasonic calls are often impaired in rodent models of autism spectrum disorders, a condition that can be comorbid with dystonia. Together, we show that these assays can serve as useful quantitative tools for investigating how neural dysfunction during development influences neonatal behaviors in a dystonia mouse model. Our data implicate a shared cerebellar circuit mechanism underlying dystonia-related motor signs and social impairments in mice.

## INTRODUCTION

Dystonia is a complex neurological movement disorder characterized by involuntary muscle contractions that can cause rigid limbs and/or twisting postures (1). These behaviors typically arise because agonist and antagonist muscles either co-contract or contract persistently, causing repetitive and sometimes sustained motions. The affected muscles can be found in a single body part as in focal dystonia or in multiple muscle groups as in generalized dystonia (2). The motor behavioral features of dystonia are often the predominant signature of the disease and are thought to reflect the underlying neural dysfunctions that cause primary dystonia. However, dystonia can also occur as a secondary symptom in neurodevelopmental conditions, neurodegenerative diseases, or acquired neurologic dysfunction. Mechanistically, this diversity is an important consideration as dystonia onset can occur in patients of all ages. Current evidence indicates that the dystonia-associated motor impairments arise from circuit deficits throughout the brain including the basal ganglia, cerebellum, thalamus, and motor cortex (3–6). Despite the rapidly growing knowledge of the genetic and circuit bases of dystonia pathophysiology, the heterogeneity and complexity of the disease has hindered a full understanding of the etiology and neural deficits that cause the debilitating dystonia-associated symptoms. Specifically, how the altered behaviors arise during development remains unclear.

There is emerging evidence pointing towards impaired neurodevelopment as a key factor in some forms of pediatric dystonia (7). Although certain hereditary childhood-onset dystonias have incomplete penetrance (DYT1 and DYT6), both manifesting and non-manifesting patients have abnormal neural circuit connectivity between multiple nodes within the motor circuit that are associated with dystonia. The main affected brain areas are thought to include the basal ganglia, thalamus, and cerebellum (8,9). Thus, while the appearance of dystonia may rely on additional molecular and circuit modifying factors (7,10,11), aberrant circuit development appears central to the behavioral expression when genetic mutations are associated with the disease. Furthermore, affected patients often display their first motor signs during childhood, whereas carriers who remain asymptomatic through childhood rarely develop symptoms later in life (12,13). This difference in behavioral onset could indicate a neurodevelopmental period during which network dysfunction leads to dystonia, coinciding with a critical developmental window that also appears relevant to other neurodevelopmental disorders including autism spectrum disorders (14). In accordance with this hypothesis, a number of the recently identified dystonia-associated mutations involve genes that are also known for their role in neurodevelopment, including the autism-associated gene, CDH8 (15–20). In addition, dystonia is also a frequent comorbidity in infants with other genetic, neurodevelopmental disorders including Rett syndrome (MECP2 mutations) (21,22), Partington syndrome (ARX mutations) (23), and as mentioned before, in an array of patients with autism spectrum disorders (24,25). Together, these studies suggest that some iterations of pediatric dystonia may emerge from aberrant neurodevelopmental processes. To test this hypothesis *in vivo*, it is crucial to investigate how behavior is shaped during development in the context of dystonia.

Multiple reports now indicate that mouse models harboring loss-of-function mutations in genes that cause the most common hereditary pediatric-onset dystonias (DYT1 and DYT6) do not display the involuntary muscle contractions or abnormal posturing observed in infants with the disorder (26–28). However, mice that have brain-restricted loss of Tor1a or knock-in mutations that mimic the human condition show several limb and body motor abnormalities that are observed in human DYT1 (29). Mouse models with overt dystonia-associated impairments can also be induced by infusing drugs into the brain (30–32) or by downregulating the expression of dystonia-associated genes (33–35) in adult mice. Electrophysiological recordings in the brains of rodent models with overt dystonic postures showed irregular burst-like firing patterns in cerebellar neurons, suggesting abnormal cerebellar function as a critical and likely shared feature of dystonia pathogenesis (30,32–34,36–39). In line with these findings, abnormal cerebellar neuron function has been confirmed in non-manifesting genetic models for DYT1 and DYT6 (40,41). Furthermore, cerebellar dysfunction is also compatible with childhood-onset dystonia of other etiologies; the protracted timeline of cerebellar development (42,43) facilitates the postnatal refinement of motor control (44,45) during a period in which even healthy infants exhibit dystonia-associated features (46–48). Indeed, we have previously found that manipulations during cerebellar development result in early-onset dystonia in mice (36,49). In one of these models, impairing excitatory synaptic transmission from brainstem inferior olive neurons onto cerebellar Purkinje neurons causes dystonia without persistent gross cerebellar malformations (*Ptf1a*^*Cre*^*;Vglut2*^*fl/fl*^ mice (36,39)), similar to what is found in infants with the disease. As a result, this engineered mouse model offers an excellent platform to investigate how aberrant neural circuit function alters neurodevelopment and subsequently impacts behavior.

However, studying dystonia in the context of neurodevelopment is often challenging. Motor control is refined during postnatal development such that even normally developing infants display high scores on the same dystonia rating scales that are commonly used to examine adults (47,48). Similarly, dystonia rating scales used to quantify dystonic movements in mature mice provide higher scores in developing control mice since the latter’s motor control is not yet refined, resulting in the reduced sensitivity of these rating scales. Another approach to quantify dystonia in animal models includes EMG recordings (34,50,51). Unfortunately, these invasive EMG recordings require surgeries and implants that are hard to perform in growing animals with small limbs. Additionally, albeit not specific to dystonia-associated impairments, global measures that are often used to assess motor control in adult mice, including the accelerating rotarod or the open field assay (31,36), cannot be easily performed with reliability and reproducibility in young postnatal developing pups because rodents at this age demonstrate less ambulatory activity and rudimentary motor skills overall.

Importantly, several behavioral assays specifically designed to assess neural function in young rodents do exist (52,53): the negative geotaxis reflex, the righting reflex, and the detection of ultrasonic vocalizations (USVs) after pups are separated from the mother (54,55). Here, using these tests, we quantify the performance of a mouse model with early-onset dystonia, the *Ptf1a*^*Cre*^*;Vglut2*^*fl/fl*^ mutant (36,39). We have chosen to focus on a key period of neurodevelopment when these motor reflexes emerge in normally developing mice, from postnatal day (P) 7–11. We found impaired acquisition of these motor behaviors in pups with dystonia, demonstrating that these paradigms may be good quantitative assays for measuring aberrant neurodevelopment in mouse models of pediatric dystonia. Furthermore, we found that the dystonic motor behaviors are accompanied by alterations in USVs. Crucially, the co-expression of motor and non-motor defects mirrors the multi-domain deficits seen in some pediatric neurodevelopmental syndromes.

## METHODS

### Animals

Mice used in this study were housed in a Level 3, AALAS-certified vivarium. Experiments and studies were reviewed and approved by the Institutional Animal Care and Use Committee (IACUC) of Baylor College of Medicine (BCM). Mice were ear-tagged on the first day of behavioral investigation (P7) and their genotypes determined using allele-specific PCR amplification after the conclusion of experiments so that experimenters were blinded to the genotypes of the mice. The day a copulation plug was detected was considered embryonic day (E) 0.5. We defined P0 as the date of birth. For the experiments in this study, we crossed male mice that were heterozygous for *Ptf1a*^*Cre*^ (JAX #023329) (56) and homozygous for the *LoxP*-flanked glutamatergic vesicular transporter 2 gene, *Vglut2*^*fl*^ (JAX #012898) (57), to female mice that were homozygous for *Vglut2*^*fl*^. The resulting *Ptf1a*^*Cre*^*;Vglut2*^*fl/fl*^ offspring had a conditional deletion of the *Vglut2* allele in the *Ptf1a* lineage, which prevents the loading of glutamate into presynaptic vesicles during fast neurotransmission and therefore eliminates neurotransmission at chemical synapses of glutamatergic, *Ptf1a* lineage neurons (58). Most *Ptf1a* lineage neurons are inhibitory neurons and therefore are not affected by the deletion of *Vglut2,* although inferior olive neurons that send climbing fiber projections to cerebellar Purkinje cells in the molecular layer of the cerebellar cortex are excitatory and do express *Vglut2*. Preventing communication between climbing fibers and Purkinje cells results in severe, early-onset dystonia-associated impairments (36,39). In our study, we used all the pups from 4 litters, which provided us with 21 *Vglut*^*fl/fl*^ control mice (7 female, 14 male) and 10 *Ptf1a*^*Cre*^*;Vglut2*^*fl/fl*^ dystonic mice (5 female, 5 male). When comparing mice from each sex and genotype with each other, we did not observe any sex differences. For this assessment, we performed a two-way ANOVA (genotype, sex) and did not find interactions between genotype and sex (*p* > 0.05 for all tests) at any of the developmental time-points for any of the behavioral tests. Therefore, we combined the data collected from male and female mice when performing the statistical analyses reported in this study.

### Negative Geotaxis Reflex

Mice were tested in the negative geotaxis assay at P7, P9 and P11 (54,59). A cage top wrapped with a sterile Poly-Lined drape was used to create a ramp with a 35° slope. Mice were placed on this ramp one at a time, oriented to face down the slope. Upon placement and release of the mouse, a 60-s timer was started. A successful trial was considered as one in which the mouse turned >90° on the ramp (crossing the plane perpendicular to the original placement in either direction). The time it took for the mouse to perform this movement was recorded. A failed trial was considered one in which mice were unable to change their orientation within 60-s or in which mice lost their footing on the ramp and fell. After a completed trial (either success or failure), each mouse was returned to their home cage. For each mouse, this process was repeated for a total of three trials per mouse, per behavioral timepoint.

### Surface Righting Reflex

The righting reflex was measured at P7, P9, and P11 (49,54). In this assay, each mouse was placed on its back on a clean cage without bedding, and then this position was gently held by one finger until timing started. Upon removal of the finger, the time required for the mouse to right itself onto its four paws was recorded. All mice were tested three times at each age. A “failed” trial was defined as one in which the mouse did not right itself within 60 s.

### Ultrasonic Vocalizations

Pup vocalizations were recorded at P7, P9, and P11 as described previously (49,60). Pups were placed in an anechoic, sound-attenuating chamber (Med Associates Inc.) within a round plastic tub that was positioned under a CM16 microphone (Avisoft Bioacoustics) in the center of the chamber. Sound was amplified and digitized using UltraSoundGate 416H at a 250 kHz sampling rate and a bit depth of 16 while Avisoft RECORDER software was used to collect the recordings. The USVs of each pup were monitored for 2 min.

### Statistical Analyses

Analyses for this study were performed using MATLAB (Mathworks, United States). We plotted and then quantified statistically significant differences using a repeated-measures ANOVA and quantified the differences between genotypes and time-points using a Tukey Kramer post-hoc analysis. We used an alpha of 0.05 to accept statistical significance.

## RESULTS

### Dystonic Postures

**FIGURE 1 |:**
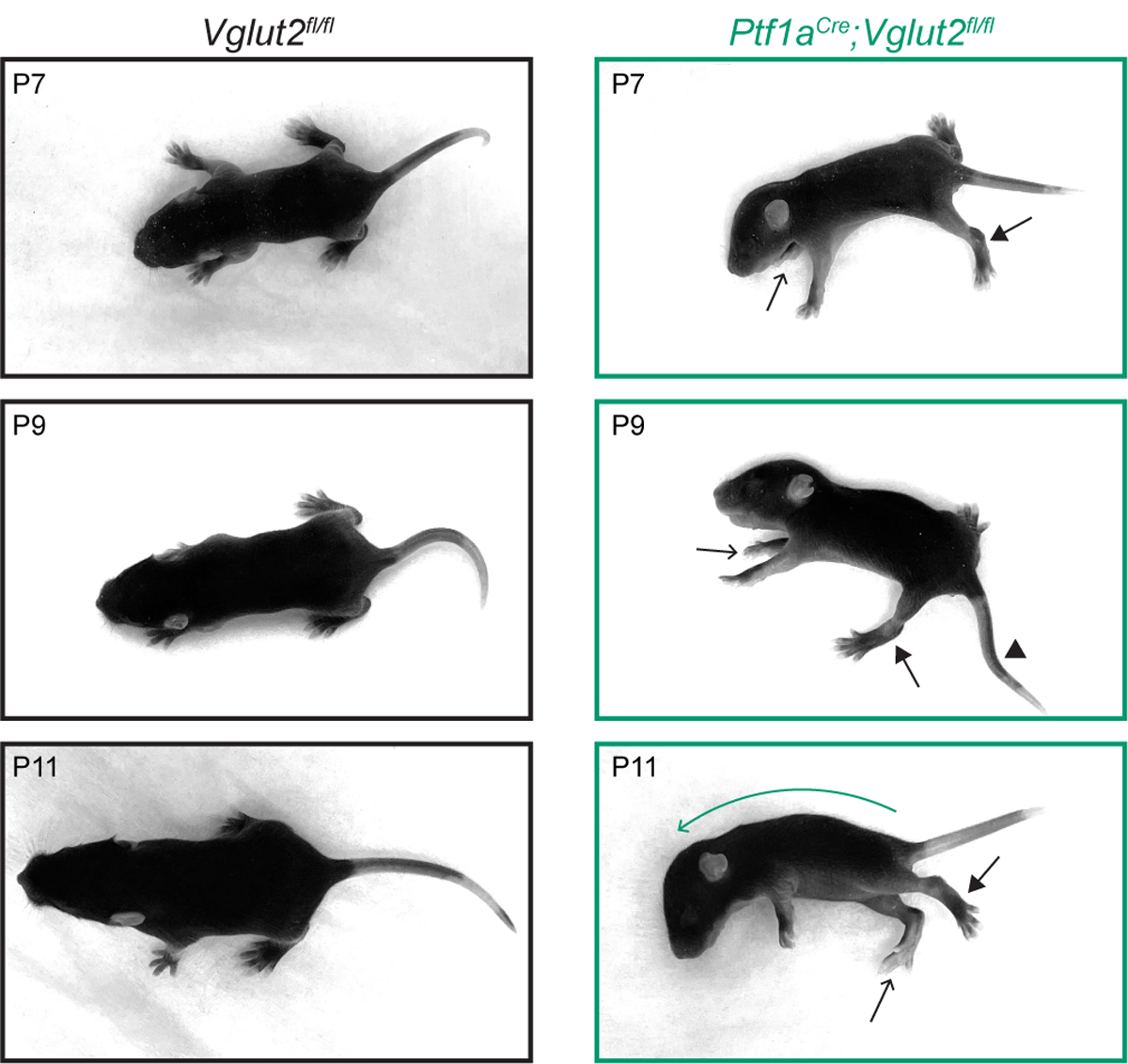
Dystonic postures in *Ptf1a*^*Cre*^*;Vglut2*^*fl/fl*^ pups. Pictures of postures in P7–P11 control and *Ptf1a*^*Cre*^*;Vglut2*^*fl/fl*^ mice. *Ptf1a*^*Cre*^*;Vglut2*^*fl/fl*^ mice have several dystonic postures, including hyper-extended limbs (arrows with closed arrowheads), twisted body posture resulting in asymmetric positioning of the paws (arrows with open arrowheads point to right paws positioned on left side of body), kink in the tail (arrowhead), and curved spine (green curved arrow, P11). These specific dystonic postures were observed in all *Ptf1a*^*Cre*^*;Vglut2*^*fl/fl*^ mice (*n* = 10).

We started by confirming the presence of dystonic postures in mice during the second postnatal week (P7-P11). At this age, both healthy control and dystonic *Ptf1a*^*Cre*^*;Vglut2*^*fl/fl*^ mice show very few coordinated, ambulatory movements, and therefore the ability to accurately distinguish spontaneous dystonic movements is difficult. Nevertheless, when positioned on a flat surface, we observed that the *Ptf1a*^*Cre*^*;Vglut2*^*fl/fl*^ pups often remained in fixed dystonic postures without initiating specific dedicated movements. To distinguish the postures seen in *Ptf1a*^*Cre*^*;Vglut2*^*fl/fl*^ mutants compared to control mice, we acquired a series of images to help visualize the different body postures displayed from P7 to P11 (Figure 1). In addition, we also relied on video recordings to examine the full extent and dynamics of the dystonia-associated behaviors (Supplementary Video S1). In the *Ptf1a*^*Cre*^*;Vglut2*^*fl/fl*^ mice, we frequently observed dystonic postures including: hyper-extension of the hindlimbs, twisted body posturing that resulted in the front- or hind-paws extending on either side of the body, strongly curved spines and lateral positioning, rigid or kinked tail positioning, and splayed digits on the fore- and hind-paws (39). Combinations of these dystonic postures were evident in all *Ptf1a*^*Cre*^*;Vglut2*^*fl/fl*^ mice (*n* = 10) included in our study (Figure 1). These data confirm that the *Ptf1a*^*Cre*^*;Vglut2*^*fl/fl*^ mutant mice have early-onset dystonia-associated motor impairments that are robust and reproducible from animal to animal.

### Negative Geotaxis Reflex

**FIGURE 2 |:**
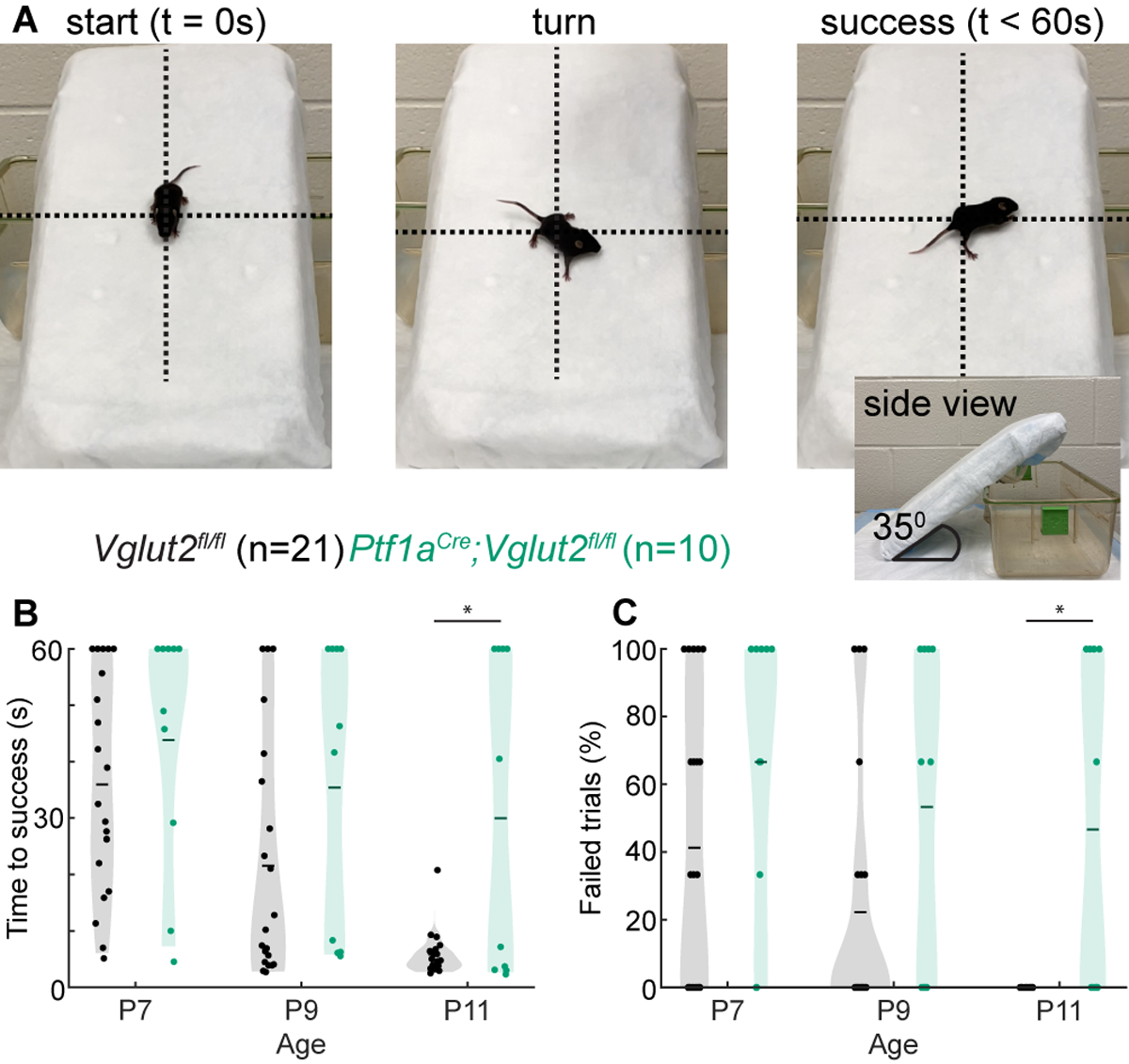
Abnormal negative geotaxis reflex in *Ptf1a*^*Cre*^*;Vglut2*^*fl/fl*^ pups. **(A)** Visualization of negative geotaxis reflex paradigm. The time to successful completion of the reflex is measured. **(B)** Average time to reflex was longer in P11 (*p* < 0.0001) *Ptf1a*^*Cre*^*;Vglut2*^*fl/fl*^ mice compared to control mice. **(C)** The number of failed trails was higher in P11 (*p* < 0.0001) *Ptf1a*^*Cre*^*;Vglut2*^*fl/fl*^ mice compared to control mice. Statistical significance was assessed using a repeated measures ANOVA followed by Tukey Kramer post-hoc analysis.

Next, we investigated whether these dystonia-associated impairments prevented *Ptf1a*^*Cre*^*;Vglut2*^*fl/fl*^ mice from properly executing the negative geotaxis reflex (Figure 2A; Supplementary Video S2). This behavioral reflex naturally arises in mice around P7 (61) and when mice mature, their time to complete the negative geotaxis reflex decreases due to their increasing motor control. We found that at the youngest ages tested (P7 and P9), control *Vglut2*^*fl/fl*^ mice and dystonic *Ptf1a*^*Cre*^*;Vglut2*^*fl/fl*^ mice took equally long to complete geotaxis reflexes (Figure 2B). However, while control *Vglut2*^*fl/fl*^ mice showed a rapid decrease in the time needed to successfully turn by P11, the time taken to turn remained prolonged in P11 dystonic *Ptf1a*^*Cre*^*;Vglut2*^*fl/fl*^ mice (Figure 2B). Similarly, we found that the number of failed trials (trials in which the pup lost grip of the padding and rolled down the slope or did not turn within 60 s) was similar between control and *Ptf1a*^*Cre*^*;Vglut2*^*fl/fl*^ mice at P7 and P9, but was higher at P11 in *Ptf1a*^*Cre*^*;Vglut2*^*fl/fl*^ mice. Together, these results indicate that, compared to controls, *Ptf1a*^*Cre*^*;Vglut2*^*fl/fl*^ mice have delays in the development of the negative geotaxis reflex, which assays the early development of motor control.

### Surface Righting Reflex

**FIGURE 3 |:**
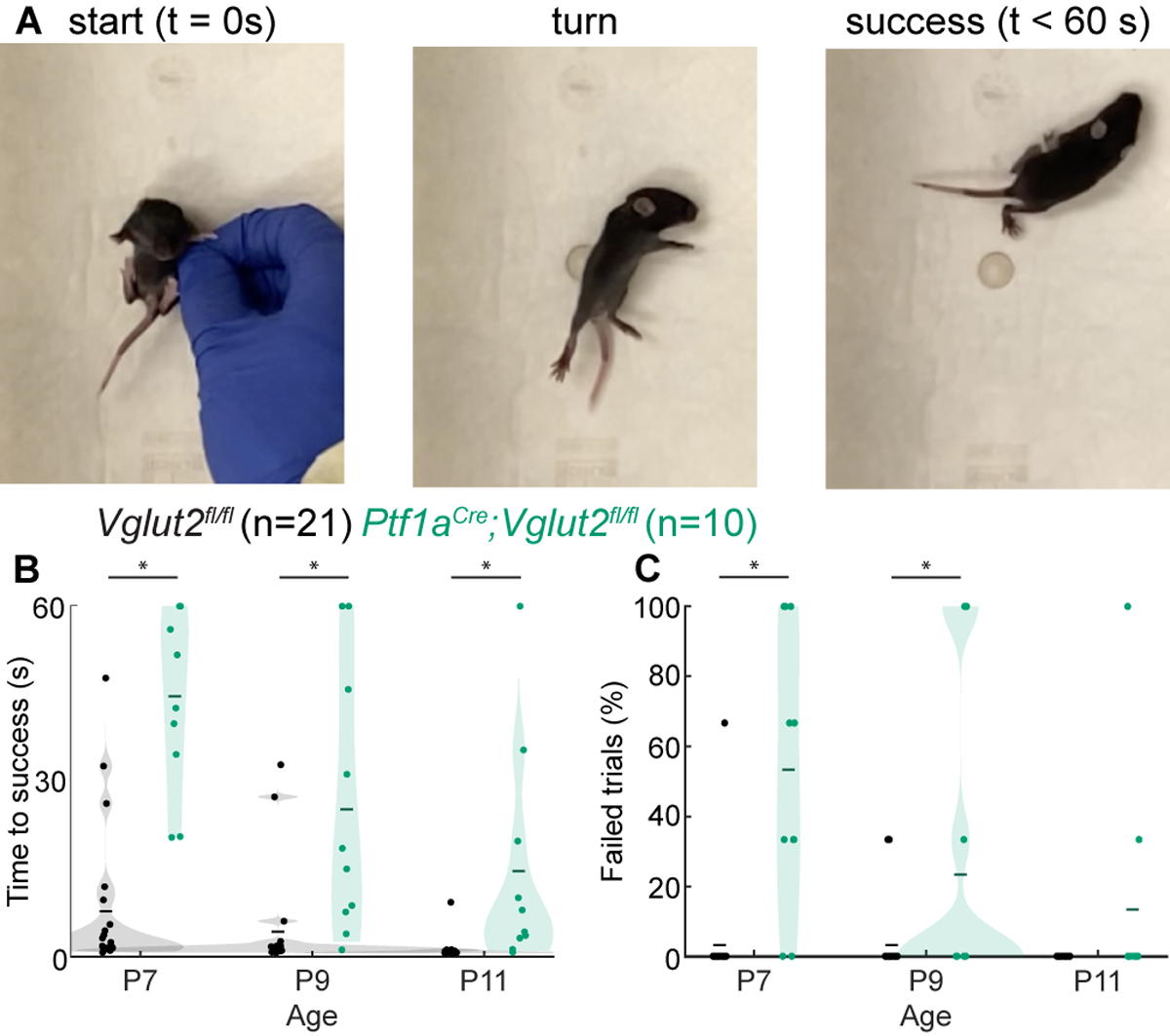
Abnormal righting reflex in *Ptf1a*^*Cre*^*;Vglut2*^*fl/fl*^ pups. **(A)** Visualization of righting reflex paradigm. The time to successful completion of the reflex is measured. **(B)** Average time to reflex was longer in P7 (*p* < 0.0001), P9 (*p* < 0.0008), and P11 (*p* = 0.0030) *Ptf1a*^*Cre*^*;Vglut2*^*fl/fl*^ mice compared to control mice. **(C)** The number of failed trails was higher in P7 (*p* < 0.001) and P9 (*p* = 0.0422) *Ptf1a*^*Cre*^*;Vglut2*^*fl/fl*^ mice compared to control mice. Statistical significance was assessed using a repeated measures ANOVA followed by Tukey Kramer post-hoc analysis.

We further investigated early motor control in dystonic *Ptf1a*^*Cre*^*;Vglut2*^*fl/fl*^ mice by examining their performance of the surface righting reflex (Figure 3A; Supplementary Video S3). Mice usually acquire this reflex around P5 (62), although some variability in reflex onset and reflex time may be observed. We previously studied *En1*^*Cre*^*;Atoh1*^*fl/−*^ mice that have impaired neurogenesis of excitatory neurons, which lead to dystonic motor impairments (49). These mice also exhibit impairments in the surface righting reflex, which was likely due to abnormal motor control. We therefore tested whether the surface righting was also impaired in dystonic *Ptf1a*^*Cre*^*;Vglut2*^*fl/fl*^ mice (Figure 3A). We found that the *Ptf1a*^*Cre*^*;Vglut2*^*fl/fl*^ mutant mice required more time to right themselves from a supine position to all four paws at P7, P9, and P11 (Figure 3B) relative to controls, although we did note that the time they took to right decreased with age. Furthermore, we observed that the number of trials in which the *Ptf1a*^*Cre*^*;Vglut2*^*fl/fl*^ mice failed to right themselves within 60 s was higher than in control mice at P7 and P9, but not at P11 (Figure 3C). Together, these results show that the *Ptf1a*^*Cre*^*;Vglut2*^*fl/fl*^ mutant mice, which express striking dystonic behaviors including bouts of twisting of the limbs and torso, also have delayed development of the surface righting reflex, likely reflecting impairments in the development of normal motor control.

### Ultrasonic Vocalizations

**FIGURE 4 |:**
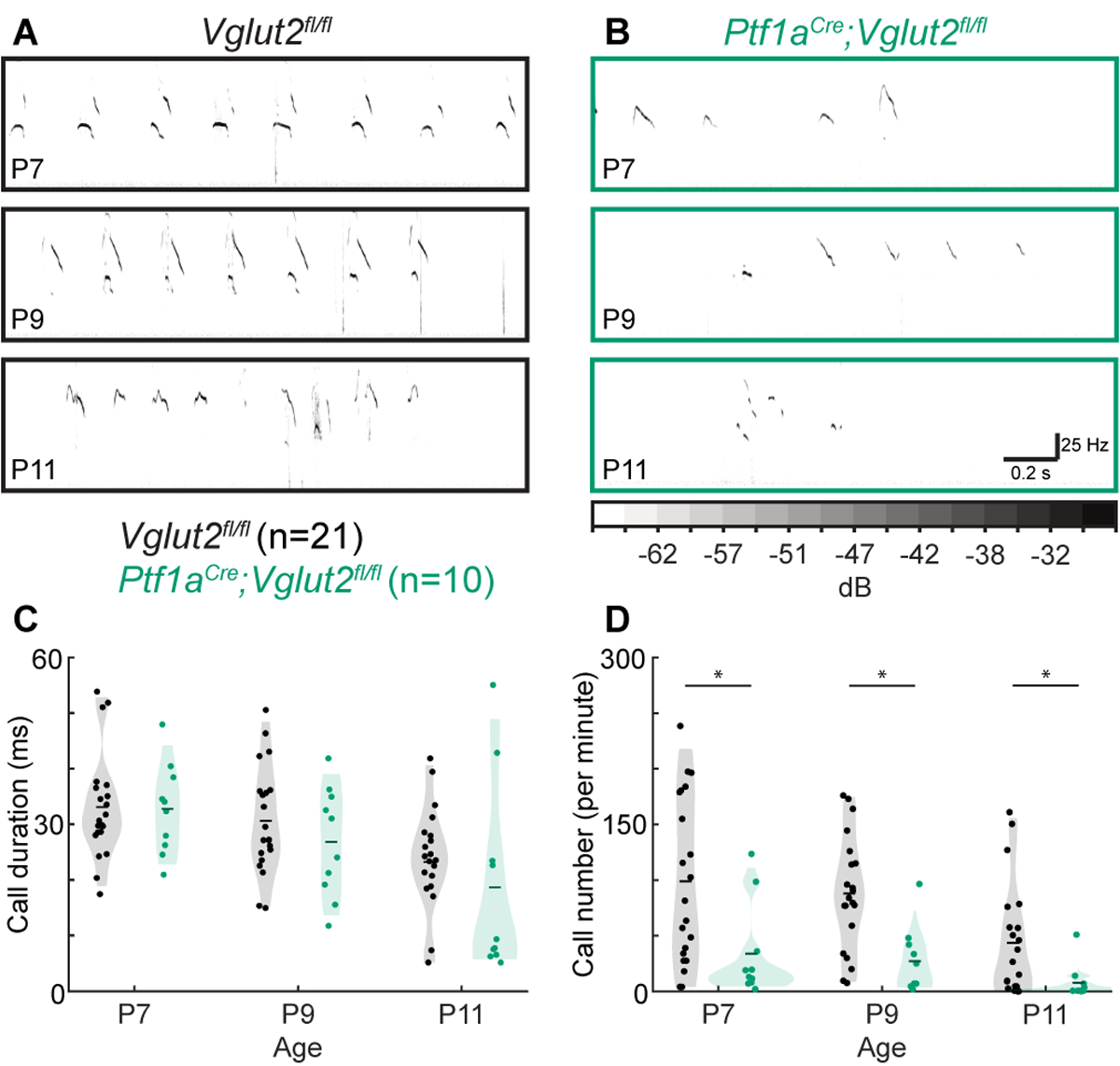
Abnormal vocalizations after separation from the dam in *Ptf1a*^*Cre*^*;Vglut2*^*fl/fl*^ pups. **(A)** Representative ultrasonic vocalizations in control pups. **(B)** Representative vocalizations in *Ptf1a*^*Cre*^*;Vglut2*^*fl/fl*^ pups. In **(A)** and **(B),** darker coloring represents louder vocalizations, scaling is the same across all figure panels. **(C**) No difference was found in the durations of calls at any time point. **(D)** The number of calls was lower in P7, P9, and P11 *Ptf1a*^*Cre*^*;Vglut2*^*fl/fl*^ mice compared to control mice. Statistical significance was assessed using a repeated measures ANOVA followed by Tukey Kramer post-hoc analysis.

To test whether the circuit disruptions in *Ptf1a*^*Cre*^*;Vglut2*^*fl/fl*^ mice lead to aberrant function in behavioral domains outside of motor control, we tested whether the *Ptf1a*^*Cre*^*;Vglut2*^*fl/fl*^ mutant mice had abnormal vocalizations when socially isolated. In the first few postnatal weeks, pups make USVs when separated from the nest and their dam (55) (Figures 4A,B). These vocalizations are thought to be a measure of early social behavior in mice and are often changed in number, duration, and/or frequency in models of neurodevelopmental disability and autism spectrum disorders (63–65). Interestingly, pup vocalizations are also abnormal in mouse models with other cerebellar alterations (49,66,67) as well as in dystonic rats (60). We found that while call duration was not statistically different between control *Vglut2*^*fl/fl*^ mice and the dystonic *Ptf1a*^*Cre*^*;Vglut2*^*fl/fl*^ mutant mice at P7, P9, or P11 (Figure 4C), the number of calls was significantly lower in *Ptf1a*^*Cre*^*;Vglut2*^*fl/fl*^ mutants compared to control *Vglut2*^*fl/fl*^ mice at P7, P9, and P11 (Figure 4D). These results show that *Ptf1a*^*Cre*^*;Vglut2*^*fl/fl*^ mice that express dystonic motor behaviors also produce fewer vocalizations of normal duration compared to littermate controls, reflecting possible deficits in socialization in addition to the observed deficits in motor control (36).

## DISCUSSION

In this study, we investigated the expression of early postnatal reflexive behaviors in the *Ptf1a*^*Cre*^*;Vglut2*^*fl/fl*^ mouse model of early-onset dystonia (36). We found that these mice have delayed acquisition of the negative geotaxis and surface righting reflexes compared to their control littermates. We further observed that the dystonic *Ptf1a*^*Cre*^*;Vglut2*^*fl/fl*^ mice make fewer USVs when separated from their dams, a behavioral impairment that is frequently seen in mouse models of neurodevelopmental disability and autism spectrum disorders. Together, we show that these simple assays can serve as a practical toolkit for investigating delays in the achievement of neurodevelopmental milestones using a circuit-based mouse model of early-onset dystonia.

The behavioral assays described in this study are particularly advantageous as they provide straightforward methods to study the impact of functional neural defects during a postnatal period that is often considered experimentally difficult to assess. The quantification of reflexive behaviors is unbiased, the assays are non-invasive, and the motor assays do not require special equipment. Furthermore, the impairments in these reflexive motor behaviors, as reported here, are not unique to rodent models of dystonia (68). For example, impairments in surface righting, but not the geotaxis reflex, have also been observed in ataxic and tremoring shaker rats (69) and ataxic lurcher mice (70). However, these reflexive behaviors are uniquely impaired in models with impaired development. For example, the neurodegeneration that occurs later in life in a mouse model for spinocerebellar ataxia 6 (SCA6) is not accompanied by impairments in either reflexive behavior (71). Due to their non-invasive nature, these postnatal reflex assays can provide quantitative and reliable measures of the relative motor impairments caused by dystonia or other developmental disorders. Such measures could be used to assess the onset of developmental motor disorders and evaluate improvements of motor behavior after providing treatment.

Regarding the behavior of *Ptf1a*^*Cre*^*;Vglut2*^*fl/fl*^ mice, we confirmed that they exhibit dystonic motor behaviors at early postnatal ages (Figure 1), making this genetic mouse model a powerful tool to study neurological deficits and circuit dysfunction in pediatric-onset dystonia. Furthermore, a recent study has shown that homozygous loss of *TSPOAP1* causes pediatric-onset dystonia through synaptic abnormalities in the cerebellum, underscoring that aberrant synaptic function in the cerebellum can also cause dystonia in humans (72). Adult *Ptf1a*^*Cre*^*;Vglut2*^*fl/fl*^ mice respond to cerebellar deep brain stimulation targeted to the cerebellar nuclei (36), making this mouse a seminal pre-clinical model to predict the efficacy of cerebellar deep brain stimulation in alleviating symptoms in patients with dystonia (73,74). Furthermore, gross cerebellar hemispheric dysfunction is a frequent finding in children born early preterm (75) of whom many suffer from dystonia (76). We therefore propose that even though the specific cerebellar network perturbation in dystonic *Ptf1a*^*Cre*^*;Vglut2*^*fl/fl*^ mice differs from those induced in other hereditary or acquired pediatric dystonias, the perturbations that impact the “dystonia network” and cause tractable dystonic motor behaviors that can be easily quantified ultimately may be relevant to and potentially predictive of the neural dysfunction(s) that are exhibited in many forms of dystonia.

Of special interest is our finding that dystonic *Ptf1a*^*Cre*^*;Vglut2*^*fl/fl*^ mice exhibit social deficits in the social isolation/USV assay. The comorbidity of social deficits, dystonia, and delayed early behavioral reflexes supports the hypothesis of a possible shared ontogeny in the domains of social interaction and motor control (43,77,78). Intriguingly, in our *Ptf1a*^*Cre*^*;Vglut2*^*fl/fl*^ mice, the resulting behaviors can be attributed to initial cerebellar dysfunction due to the precision of the genetic manipulation performed in these mice (36). The observed USV changes further suggest that neurodevelopmental disorders exhibiting deficits across developmental domains, as seen in dystonia, may converge on a shared mechanism of cerebellar dysfunction. Previous work has highlighted the importance of evaluating motor control in mouse models of autism spectrum disorders and cerebellar dysfunction in patients with autism spectrum disorders to uncover the full range of neural dysfunction in these neurodevelopmental disorders (63,79,80). Here, we propose the reverse; in pediatric dystonias and other early-onset movement disorders, social deficits should be taken into consideration and fully evaluated. In conclusion, we postulate that neurodevelopmental disorders that display a spectrum of social and motor deficits may converge at the level of cerebellar dysfunction (42,43,77,81). It would be interesting if future studies were to investigate whether the manifestation of motor and social impairments in dystonia is dependent on region-specific dysfunctions (80), how the resulting dysfunction is related to the underlying genetic mechanisms, and whether the ultimate behavioral abnormalities rely on the timing of genetic or physical insults to the developing cerebellum and its associated brain networks.

## Supplementary Material

Video S3 **Supplementary Video S3 |** Surface righting reflex. The *Vglut2*^*fl/fl*^ pup immediately turns from the supine position onto its four paws, whereas the *Ptf1a*^*Cre*^*;Vglut2*^*fl/fl*^ pup takes longer to right itself.

Video S2 **Supplementary Video S2 |** Negative geotaxis reflex in P11 mice. The *Vglut2*^*fl/fl*^ pup immediately turns to face upwards, whereas the *Ptf1a*^*Cre*^*;Vglut2*^*fl/fl*^ pup takes longer to turn towards the upward direction.

Video S1 **Supplementary Video S1 |** Ambulatory activity in control and dystonic P9 mice. Dystonic motor signs are evident in the *Ptf1a*^*Cre*^*;Vglut2*^*fl/fl*^ pups, but note that movements are also relatively uncoordinated in control *Vglut2*^*fl/fl*^ pups.

## Data Availability

The raw data supporting the conclusion of this article will be made available by the authors, without undue reservation.
